# Cognitive Behavior Therapy (CBT) and Positive Psychotherapy (PPT) for the treatment of anxiety disorders in an online group setting: A randomized controlled trial

**DOI:** 10.1371/journal.pone.0355614

**Published:** 2026-08-12

**Authors:** Catiana L. Engelhardt, Sabrina Keller, Thomas Berger, Anton-Rupert Laireiter

**Affiliations:** 1 Department of Psychology, Paris Lodron Universität Salzburg, Salzburg, Austria; 2 Institute of Psychology, University of Bern, Bern, Switzerland; South China Normal University, CHINA

## Abstract

**Objective:**

The goal of this randomized controlled trial is to evaluate the efficacy of PPT compared to CBT and to test whether PPT is as effective or even more effective than CBT in reducing anxiety symptoms and improving the patients’ quality of life. The interventions last ten weeks and takes place in an online group setting.

**Methods:**

A total of 110 patients were divided into two intervention groups (PPT: *n* = 53; CBT: *n* = 57). The measurements were conducted using online questionnaires to evaluate the anxiety, subjective happiness and quality of life (primary outcomes), and the levels of psychological distress (secondary outcome). These measures were obtained at three time points: the beginning of the study (T0), the end of the study (T1), and the follow-up three months later (T2).

**Results:**

The results for the main effect group were not significant, with one exception for the Fear Questionnaire (FQ). The time × groups interaction were not significant. The time factor consistently showed medium to high effects sizes. The levels of anxiety, subjective experience of happiness, and psychological distress improved significantly from pre- to post-treatment in both intervention groups, with effects persisting until the three-month follow-up.

**Conclusion:**

Our study could show that the positive psychology intervention yields the same effects as the standardized CBT for the treatment of anxiety disorders, as there were no significant differences between the two treatment groups in terms of reduction of anxiety symptoms and improvement in satisfaction and quality of life over time.

## Introduction

Anxiety disorders are severe in intensity and tend to become chronic [1], interfering with everyday activities, debilitating the affected person and thus, reducing their quality of life. Based on the updated S3-guideline for the treatment of anxiety disorders, the general recommendation for generalized anxiety disorder, panic disorder, agoraphobia, and social phobia is either psychotherapy (the first-line treatment being Cognitive Behavioral Therapy, CBT) or pharmacotherapy [2]. A combination of both options (e.g., CBT and SSRIs) can be considered if one form of therapy alone is not effective enough.

As an alternative, other therapeutic approaches such as Positive Psychotherapy (PPT) are worth considering. PPT is a relatively new approach derived from positive psychology, which focuses on finding ways to increase well-being and build a positive self [3]. Its therapeutic process is built on three key elements: (1) the human desire to grow and live a fulfilled, happy life, (2) understanding the importance of positive resources instead of focusing on the negative, and (3) building a good therapeutic alliance by steering the interaction towards positive aspects of the patient’s life [4]. PPT was originally founded by Nossrat Peseschkian, who stated that “positive psychotherapy focuses not on the problem, but on the person; not on deficits, but on resources” [5]. He emphasized that every individual possesses inner resources and potential for growth, and therapy should help uncover and strengthen these capacities. Later, Martin Seligman, widely regarded as the father of positive psychology, described the integration of positive psychology into psychotherapy as a way to “build what’s strong, not just fix what’s wrong” [6]. This perspective shifted the focus from a deficit-oriented to a strength-based approach, aiming to foster positive emotions, engagement, relationships, meaning, and accomplishment (PERMA model) [7]. Additionally, Mihaly Csikszentmihalyi contributed to the understanding of optimal experience through his concept of flow, describing it as “the state in which people are so involved in an activity that nothing else seems to matter” [8]. Flow experiences are integral to PPT as they help clients identify and cultivate their personal strengths and passions. Thus, PPT is a psychotherapeutic method which puts emphasis on increasing positive emotions, accomplishments, engagement, meaning, and wellbeing, all while also addressing and working on concerns and problems. By building personal resources, difficult situations can be coped with better, and quality of life can be improved [9].

The integration of positive psychology and psychopathology to find new ways of treating mental disorders has been studied mostly by using positive psychology interventions (PPIs). Fairly recent research on PPT and PPIs has shown its effectiveness on people with depression [10–12]. A meta-analysis of PPIs on the treatment of depressive symptoms as well as diagnosed clinical depression showed that PPIs led to significant improvements of the depression scores compared to control groups in all but one study [13]. These results were found for pre- to post- as well as pre- to follow-up measurements. [14] conducted a meta-analysis on the efficacy of PPIs and reported that, although these interventions increased well-being, the results regarding the treatment of depression were ambiguous (with rather small effect sizes) and for the most part not significant. [15] conducted a study with a larger sample size in which they compared PPT to CBT for people with depressive disorders (mild to moderate in severity) and reported a better outcome on the depression scores for patients in the PPT group compared to the CBT group. Considering the high comorbidity rate of depression and anxiety [16,17], PPT might be useful as a treatment option for anxiety as well. The latest research shows similar promising results to depression treatment for individuals treated for anxiety symptoms, with PPI offering therapeutic benefits [18] and leading to significant improvements on the anxiety score at post and follow-up measurement points compared to pre-intervention [19]. In summary, these results suggest that PPT might be applicable for the treatment of anxiety.

Although face-to-face therapy remains standard, e-therapy —where therapist and client connect via electronic means (e.g., email, video call)— has gained popularity, particularly due to factors like geographical distance, mobility issues, or severe disorders [20]. Initially met with hesitation from both therapists [21] and clients [22] due to concerns over confidentiality and liability, the COVID-19 pandemic demonstrated its importance. Studies show e-therapy’s efficacy for conditions such as anxiety and depression [23–25], OCD [26], weight loss [27], alcohol misuse [28], and smoking [29]. Internet-based CBT (iCBT) mirrors face-to-face CBT content but is delivered digitally. Research highlights iCBT’s effectiveness for alcohol dependence [30], bulimia nervosa [31], PTSD [32], depression [33,34], and anxiety [35–37]. The Ontario Health Technology Assessment [38] found significant symptom improvements in iCBT participants compared to waiting lists. Online Positive Psychotherapy (PPT) and Positive Psychology Interventions (PPIs) have also demonstrated benefits. Studies show reduced stress, anxiety, and depression [39,40], including in Greek healthcare professionals [41]. PPIs such as gratitude, self-compassion, and strengths-based exercises significantly improved students’ well-being over eight weeks [42], suggesting PPT and PPIs can be valuable additions to standard treatments for depression and anxiety.

## Materials and methods

The goal of the present study was to find an alternative to CBT in the treatment of anxiety disorders, considering that some people might not respond to the treatment or prefer a different therapeutic approach that focuses on resources and strengths. Ultimately, diversity of therapy interventions for patients with anxiety helps to provide more patient-tailored approaches. Additionally, our interest lies in the longevity of PPT treatment for anxiety compared to CBT, as this determines whether PPT should be seriously considered as a treatment option for anxiety disorders. Thus, a three-month follow-up is implemented to evaluate the durability of the effect of the treatment.

Based on the existing research literature, we hypothesize PPT to lead to a significant reduction of anxiety symptoms as well as psychological distress and a significant increase of subjective feeling of happiness and quality of life pre- to post-therapy. This improvement in all measurement outcomes should persist from post- to follow-up measurement. These results in the PPT group are hypothesized to be similar to the outcome of the CBT group.

### Study design and protocol

**Fig 1:**
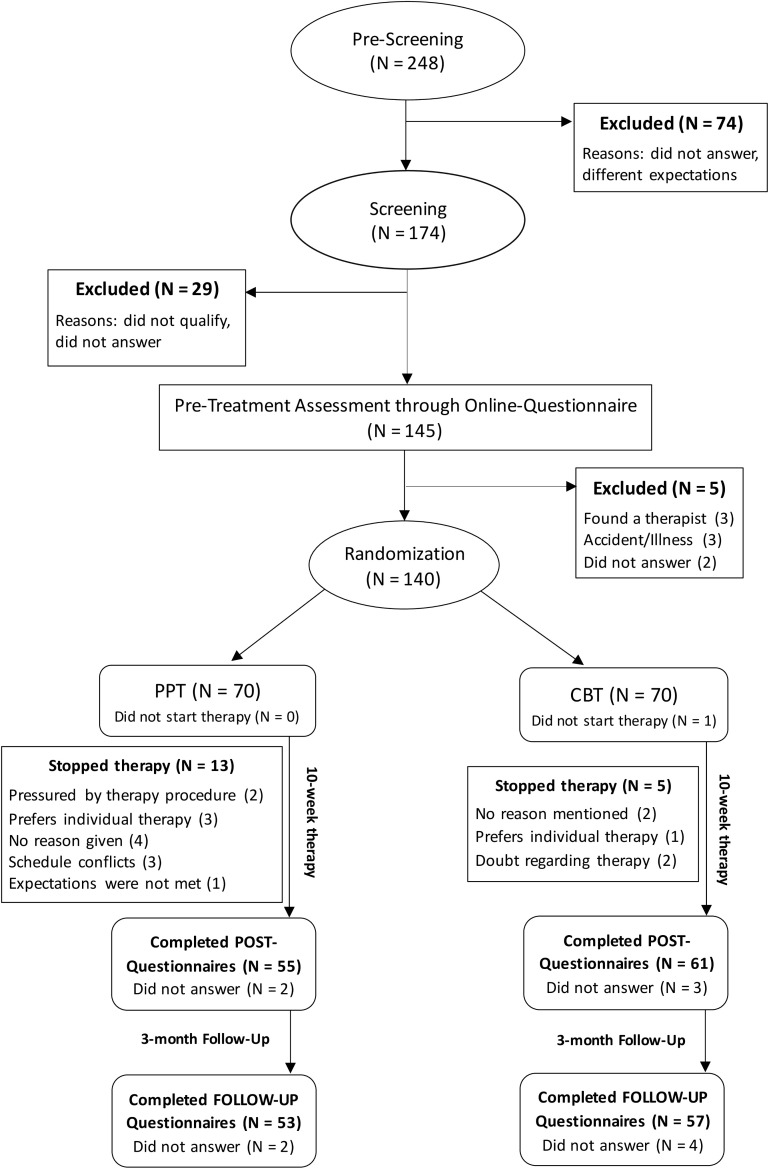
Flowchart of the study design.

The work presented here, is part of a larger series of studies reported in the study protocol [43]. A three-arm randomized controlled trial comparing CBT, cPPT (complete therapeutic supervision), and mPPT (minimal therapeutic supervision) was conducted. This manuscript focuses on the efficacy comparison between the CBT intervention group and the PPT intervention group, while the comparison between the PPT with complete therapeutic support and the PPT with minimal therapeutic support is the subject of a different publication. The randomized-controlled study took place over a period of ten weeks in an online group therapy setting with the treatment conditions: PPT and CBT, to which the subjects were randomly assigned, with the help of the online platform randomizer.org. To test the efficacy hypotheses, three measurement points were implemented: the same online questionnaire was filled out by the subjects before the start of the study (PRE measure, T0), after the completion of the therapy (POST measure, T1), and at follow-up three months after end of treatment (FOLLOW-UP measure, T2). This results in a 2×3 between- and within-subjects design. Therapy for the two intervention groups was conducted in a dual therapist approach, meaning two therapists were assigned to each intervention group and were leading the therapy together. This was done to effectively respond to the participants’ questions or concerns and to give full attention to someone in a moment of crisis while being able to continue with the treatment session as intended. Also, since the therapy was carried out online, technical problems were always a possibility, which was why two therapists posed less risk of the therapy not taking place due to, e.g., connection failure. Additionally, to allow for a better psychological treatment, supervision and intervision took place at regular intervals to address questions or challenges of the therapists. Fig 1 shows the flowchart of the study. As can be seen, a total of *n* = 248 people completed the pre-screening, where contact information and consent to participate was queried and the general qualification for the study was assessed (74 participants did not stay in contact during or after that). Following the pre-screening, a more thorough screening of *n* = 174 people was held online over Zoom® (Zoom Communications; San José CA, USA) to evaluate whether the criteria for the participation in the study were met. After the screening procedure, a total of *n* = 140 people took part in the study and were randomly allocated to either the PPT or the CBT intervention group. From then on, ten weeks of therapy were implemented in an online group setting. During the course of these ten weeks, 13 people stopped participating in the PPT group, whereas five people stopped the therapy in the CBT group. Reasons were –among others– feeling stressed by the therapy, wanting to go into individual therapy or the feeling that the therapy was not working for them. This lead to 55 people completing the questionnaire after the ten weeks of therapy in the PPT group, and to 62 people completing the post-therapy questionnaire in the CBT group. The questionnaire for the follow-up three months later was filled out by 54 people in the PPT group and 59 people in the CBT group, since some participants could not be reached. The study protocol as approved by the ethics committee is available as supporting information, see S1 Appendix for the German original and S2 Appendix for the English translation. The supporting information also includes the approval letter of the ethics committee in the original language (German) S4 Appendix as well as the English translation thereof S5 Appendix. Ethical approval for this study series was granted by the Ethics Committee of Paris Lodron University Salzburg (Approval No. EK-GZ 22/2021). Verbal informed consent was obtained from all participants for the use and publication of anonymized data. The Ethics Committee approved verbal consent as appropriate for the online study format, where participants were not physically present and participation posed minimal risk. The study is registered at the German Clinical Trials Register № DRKS00027521. The implementation of the study was made possible by financial contributions from the University of Salzburg. This includes the payment of the study assistants, supervision and therapists. The authors declare no conflict of interest. The funders had no role in the study design, data collection, analyses, or in the writing. The CONSORT checklist is available as supporting information S3 Appendix. The complete raw data collected during the study are available as supporting information file under S1 Dataset.

#### Inclusion and exclusion criteria.

All study participants had to fulfill the following inclusion criteria: (1) Participants had to be between 18 and 65 years old, (2) exhibit sufficient intellectual capacity as well as command of the German language, and (3) suffer from one of the following disorder according to ICD-10: F41.0 panic disorder with and without agoraphobia and/or F41.1 generalized anxiety disorder and/or F40.1 social anxiety disorder. Intellectual capacity was assessed during a structured screening interview conducted by a psychologist, who evaluated participants’ ability to understand study procedures and instructions, engage adequately in conversation, provide coherent responses, and give informed consent, without the use of standardized psychometric or neuropsychological testing. These three disorders were selected intentionally because the corresponding exposure exercises could be implemented in a standardized and feasible manner within the online group setting. Other anxiety disorders, particularly specific phobias and agoraphobia without panic disorder, were deliberately excluded, as their treatment often requires more individualized exposure procedures and specific environmental conditions. In addition, a relatively homogeneous group composition was considered important to facilitate meaningful peer exchange and mutual learning among participants with comparable symptom presentations. In addition to the abovementioned inclusion criteria, the participants had to be ruled out if they were in concurrent or close future participation in psychological or psychotherapeutic treatment during the following three months away from beginning of the treatment. They were also excluded from the study if they suffered from any of the following disorders: major depressive episode, bipolar affective disorder or mania (current or previous), schizophrenic or schizoaffective disorder, chronic grief reaction, severe anorexia or bulimia, substance dependence (alcohol, illicit drugs), severe personality disorder. Acute suicidality was an exclusion criteria as well as a change in medication, in dose, or the complete discontinuation of the drug in recent times or the next three months.

#### Participation recruitment process.

Recruitment commenced by distributing flyers in pharmacies, doctors’ and psychotherapists’ offices, and other public buildings (e.g., universities, supermarkets, churches). Additionally, the study was promoted on social media such as Facebook and Instagram. The recruiting process began on March 8^th^, 2022 and ended on November 3^rd^, 2022. All subjects interested in participating underwent an online questionnaire pre-screening, and if qualified, had a screening appointment over a video call. To check for inclusion and exclusion criteria and reach a diagnosis, the German version of the short form of the Diagnostic Interview in Psychological Disorders (MINI-DIPS; [44]) and the German version of the Personality Disorder Screening – Short Form (PSS-K; [45]) were applied. The MINI-DIPS was conducted as a structured clinical interview, whereas the PSS-K was administered as a self-report screening instrument and subsequently reviewed within the clinical screening procedure. All participants were informed about the process and content of the study as well as the data collection procedure and had to give their verbal consent as a prerequisite for participation in the study. Verbal consent was documented with the date and time during the screening.

#### Study sample.

As described in [43], the total number of participants was calculated with the power analysis tool G*Power 3.1 [46]), assuming an alpha error of .05 and a power of ≥.80. Effect size estimates were based on findings from previous studies [47,48]. As a high dropout rate was anticipated, a considerably larger number of participants were screened and included in the initial recruitment phase. Initially, a repeated-measures mixed ANOVA was considered for the analysis. However, based on methodological considerations and to allow for greater flexibility in handling missing data and covariates, we ultimately chose to use linear mixed-effects models (LMM). Since in this article, we report the comparison between the two intervention groups (CBT and PPT), the sample size is reduced to 110. The study enlisted predominantly female participants, constituting 107 individuals (97.3%) out of the final sample size of 110 participants. The average age stands at approximately 34.41 years, with a standard deviation of 8.70 years. The age of the participants ranges from 19 to 61 years. Table 1 provides demographic profiles as well as primary and secondary outcome measures of the study sample at baseline level. Significance tests were conducted to examine possible divergences in demographic traits and baseline levels of patient-reported measures, which encompass factors such as fear, depression, happiness, and other relevant metrics. Notably, no statistically significant differences in the demographic characteristics age and gender, were identified between the two treatment groups. Furthermore, an examination of baseline levels across outcome variables revealed no significant disparities between the groups except for the Fear Questionnaire (FQ) with a mean of 21.64 (SD=9.30) in the PPT group and a mean of 17.26 (SD=9.98) in the CBT group, yielding a *p*-value of 0.019. To examine the potential influence of the observed baseline difference in FQ scores on the primary findings, a sensitivity analysis was conducted in which baseline FQ severity was included as a time-invariant covariate in the mixed-effects model, restricted to post-treatment and follow-up assessments. Baseline FQ severity significantly predicted subsequent outcome, $F(1,104.32)=94.74,p<\mathrm{.001},\eta^{2}=\mathrm{.372}$. The group × time interaction remained non-significant, *F*(1, 108.00) = 0.76, *p* = .386, indicating that explicit adjustment for baseline severity did not alter the substantive conclusions of the primary analysis (see Table 2).

**Table 1:** Descriptive statistics for demographic, outcome variables and significance tests at baseline.

	Treatment group		Total	
	PPT (n=53)	CBT (n=57)	(n=110)	Statistics
**Demographics**				
Gender: *n*(%)	Female: 52 (98.1)	Female: 55 (96.5)	Female: 107 (97.3)	$\chi^{2}$(1)=.272, p = 1.000
Age: Mean (SD)	34.62 (8.22)	34.21 (8.66)	34.41 (8.70)	t(108)=.247, p=0.805
**Primary outcomes**				
**Level of anxiety**				
FQ	21.64 (9.30)	17.26 (9.98)	19.37 (9.87)	t(108)=2.375, p=0.019
GAD7	12.98 (4.08)	11.75 (4.07)	12.35 (4.11)	t(108)=1.577, p=0.118
BAI	27.96 (11.20)	27.47 (11.54)	27.71 (11.33)	t(108)=.225, p=0.822
PAS	22.92 (9.90)	20.95 (10.45)	21.90 (10.19)	t(108)=1.017, p=0.311
**Subjective experience of happiness**				
PPTI	86.92 (14.26)	89.37 (12.80)	88.19 (13.52)	t(108)=-.947, p=0.346
FS	38.64 (8.87)	39.51 (8.60)	39.09 (8.70)	t(108)=-.521, p=0.604
SWLS	20.28 (6.37)	21.39 (6.21)	20.85 (6.28)	t(108)=-.919, p=0.294
**Secondary outcomes**				
**General psychological distress**				
ISR	46.08 (14.88)	40.96 (13.35)	43.43 (14.27)	t(108)=1.899, p=0.060
PHQ9	11.49 (5.41)	10.09 (5.02)	10.76 (5.24)	t(108)=1.411, p=0.161

FQ: Fear Questionnaire, GAD7: Generalized Anxiety Disorder, BAI: Beck Anxiety Inventory, PAS: Panic and Agoraphobia Scale, PPTI: Positive Psychotherapy Inventory, FS: Flourishing Scale, SWLS: Satisfaction with Life Scale, IRS: ICD-10 Symptom Rating, PHQ9: Patient Health Questionnaire.

**Table 2:** FQ – Sensitivity (Baseline adjusted).

Factor	df1	df2	*F*	*p*	$\eta^{2}$	$\eta_{w}^{2}$
Group	1	45.81	3.60	0.064	0.022	0.003
Time	1	108.00	0.01	0.926	0.001	0.003
Group × Time	1	108.00	0.76	0.386	0.002	0.007
FQ Baseline	1	104.32	94.74	<.001	0.372	–
Gender	1	103.02	0.31	0.578	0.002	–
Age	1	104.78	1.33	0.251	0.008	–

$\eta^{2}$: eta-squared (total); $\eta_{w}^{2}$: eta-squared (within-person). Model restricted to post-treatment and follow-up assessments. Baseline FQ included as time-invariant covariate. Reference categories: PPT (group), post-treatment (time).

### Interventions and treatment approach

The interventions were manualized for both groups, and workbooks were developed for the study participants. The PPT manual was based on the official manual by [4]. The PPT manual developed for the study was shortened from the original 15 sessions to ten sessions, and exercises from the study leader’s own PPT repertoire were added. The CBT manual was adapted from the German language therapy tools for anxiety disorders by [49]. As explained in Tables 3 and 4, the material covered in the respective intervention groups differed by session. However, every session had the same structure, starting with a quick overview on what was to be expected that week followed by psychoeducation and worksheets with exercises. Some exercises, such as gratitude journaling for the PPT group and the relaxation exercise “Progressive Muscle Relaxation” according to Jacobson for the CBT group, were instructed to be completed daily. Over the next ten weeks, participants in the PPT group learned about the impact of positive emotions, how to increase happiness inducing moments, and were introduced to the effect of gratitude. They gained more insights about their own character strengths, including core strengths, and learned ways to build and improve their own resources. Study participants in the CBT group got to reflect on their own anxiety response and the vicious cycle of fear. They learned adaptive coping strategies for stressful situations and practiced cognitive restructuring. The treatment closed out with an exposure exercise and the creation of a resource kit in order to successfully handle future anxiety-raising situations. In addition to the workbook, the participants received a link to an intervention-tailored website (created by Webflow) containing videos on the topic of each week and on the information given in the workbook. Subjects in both intervention groups (PPT & CBT) met their therapists once a week for a 120-minute online meeting, in which the sessions were conducted according to the manual. For both treatment conditions, a key component was grouping two clients within each therapy group together. The pairing was done at the beginning of the therapy and persisted for ten weeks until the end of the therapy, unless one client had a reason to change teams (e.g., dropout). In these teams, the participants had the opportunity to discuss weekly homework and were encouraged to support one another. On which communication device (e.g., video call, phone call, in person) this exchange took place was to be decided by the participants.

**Table 3:** A breakdown of the various treatment sessions included in the PPT intervention.

Treatment Sessions	Content
Session 1: Introduction	Learning about the therapy procedure, the workbooks, and the gratitude diary; finding a team partner HW: filling out the diary daily; discussing the positive psychotherapy inventory
Session 2: Positive Emotions	Knowledge about the most important positive emotions; learning strategies to increase moments of happiness HW: reflecting on one’s own emotions and in which situations those are triggered
Session 3: Gratitude	Learning about the use of gratitude and gratitude interventions HW: integrating gratitude more strongly into daily life
Session 4: Character Strengths	Introduction about character strengths, learning about your own strengths and how to effectively use them HW: filling out self-assessment work sheets and comparing those to how friends and family see you to find out your character strengths
Session 5: Core Strengths	Knowing the value of core strengths and how to regulate them; developing an individual plan of action for daily anxiety-inducing situations HW: realizing that plan in everyday life
Session 6: Static & Dynamic Self	Information about what is static and dynamic perception of self; re-interpretation of previous experiences using the dynamic self-image HW: working on dynamic and static self in depth through work sheets
Session 7: Optimism	Advantages of being optimistic and learning about as well as identifying one’s own attributional styles HW: introspection during daily life and practice having an optimistic attitude
Session 8: Self-Compassion	Introduction to as well as interventions to increase self-compassion HW: find situations in which one is strict to oneself and find way to be more lenient with oneself; practicing mediation exercises
Session 9: Resilience	Learning about the importance of resilience and ways to enhance it; building an individual resource kit HW: work on the resource kit and reflect upon the therapy
Session 10: Everest-Goals	Gain knowledge about the necessity of setting goals and identifying one’s own Everest goals HW: visualizing one’s own best positive self

HW: homework.

**Table 4:** An overview of the individual treatment session provided in the CBT intervention.

Treatment Sessions	Content
Session 1: Introduction	Learning about the therapy procedure, the workbooks and about anxiety including its purpose; finding a team partner HW: performing Progressive Muscle Relaxation daily; filling out work sheets
Session 2: The 3 Levels of Anxiety	Information about the 3 levels of anxiety and finding out one’s own anxiety response HW: practicing interventions to regulate anxiety
Session 3: Vicious Circle of Fear	Knowledge about the vicious circle model of anxiety and application of said model on the different anxiety disorders HW: Introspection to find ways to break the vicious circle in everyday life
Session 4: My Individual Explanatory Model	Learning about risk factors for anxiety disorders and the Vulnerability-Stress-Model HW: creating an individual explanatory model and practicing attention fixation by guiding one’s perception during anxious moments
Session 5: Mindfulness	Learning about coping strategies for different stress levels and mindfulness as a strategy against anxiety HW: performing and reflecting on various mindfulness exercises
Session 6: Cognitive Restructuring	Information about techniques to deal with catastrophic thinking HW: filling out work sheets to become aware of situations that trigger anxiety
Session 7: Handling Stressful Thoughts	Learning about interventions to handle negative emotions in a healthy manner HW: filling out work sheets regarding the learned interventions and applying them in daily life
Session 8: Exposure Preparation	Information about the role and efficacy of exposure, identifying one’s own avoidance strategies and preparation for the exposure in the next treatment session HW: compiling stressful situations and rating how anxiety-inducing they were
Session 9: Exposure	Exposure implementation under therapeutic guidance and independent execution of exposure exercises HW: repeatedly carrying out the exposure exercise and observing one’s own anxiety response
Session 10: Relapse Prevention	Reflecting on the therapy process and figuring out ways to handle future stressful situations in a healthy way HW: creating a resource kit

HW: homework.

### Outcomes measures

The primary outcome measures are the extent of anxiety (BAI, PAS, GAD-7, FQ) and subjective experience of happiness (PPTI, FS, SWSL), whereas the secondary outcome measure is the level of general psychological distress (ISR & PHQ-9). Both primary and secondary outcome variables are based on self-reports and measured as follows.

The Beck Anxiety Inventory, German version (BAI; [50]) is a self-assessment questionnaire containing 21 items to evaluate the level of anxiety in adolescents and adults during the last seven days using a four-point Likert scale. BAI is a very reliable test (Cronbach’s α=.91; [51]) and correlates highly (*r* = .64) with the Hamilton Anxiety Rating Scale (HAM-A; [52]). The Panic and Agoraphobia Scale, German version (PAS; [53]) assesses the severity of symptoms of the patient over the last week based on 13 items regarding five aspects that impact the quality of life (e.g., panic attacks, agoraphobic avoidance, health concerns) with a five-point Likert scale. The test is reliable (Cronbach’s α=.86) and valid (*r* = .91 with the Clinical Global Impression Scale (CGI; [53]). The Generalized Anxiety Disorder 7, German version (GAD-7; [54]) is an economic questionnaire consisting of seven items regarding physical and psychological anxiety symptoms to report the frequency of the symptoms over the last two weeks on a four-point Likert scale. The test has high internal consistency (Cronbach’s α=.92) and the validity of the test is shown by the high correlation with the BAI (*r* = .72). The Fear Questionnaire, German version (FQ; [55]) is a questionnaire consisting of five items to assess the extent of avoidance behavior for situations that cause anxiety (e.g., getting stared at or criticized, talking to people of authority, eating with other people) based on a nine-point Likert scale. All coefficients of internal consistency are moderate, and all scales have shown a moderate construct validity [56].

The Positive Psychotherapy Inventory, German version (PPTI; [4]) is a 25-item test that assesses the five aspects of well-being based on the PERMA theory of well-being (e.g., positive emotions, engagement, relations, meaning, achievement) on a five-point Likert scale. The questionnaire is reliable (Cronbach’s α=.89) and has a convergent validity of *r* between .50 and .70 with the Ryff’s Psychological Well-Being Scale (PWB). The Flourishing Scale, German version (FS; [57]) evaluates subjective psychological well-being based on eight items with a seven-point Likert scale. The testing instrument is reliable (Cronbach’s α=.79) and valid (moderate correlation of *r* = .57 with the Short-Form Health Survey (SF-12; [58]).

The Satisfaction with Life Scale, German version (SWLS; [59]) assesses one’s satisfaction with life consisting of five items based on a seven-point Likert scale. The SWLS shows adequate internal consistency (Cronbach’s α=.87; [60]) and is moderately correlated with the Life Satisfaction Index (*r* = .46; [59]). For a third-party assessment through the therapists on the extent of anxiety, a self-designed questionnaire was used (the dimensions were tension, anxious mood, fear, sleep disturbance, depressiveness, anxious behavior, physical symptomatology, psychological distress, impairment in living, life satisfaction, motivation & energy). Questions such as “To what extent does the patient show/report an anxious mood?” and “How impaired does the patient appear to you in his/her lifestyle due to his/her psychological stress?” were used to assess the aforementioned topics. While creating the items of the test, inspiration was drawn from questionnaires such as the PPTI, FS, and SWLS.

The ICD-10 Symptom Rating, German version (ISR; [61]) is used to evaluate the severity of the psychological symptoms based on 29 items categorized into six subscales (depression, anxiety, obsessive-compulsive, somatization, eating disorder, and an additional scale with individual items with screening function for individual syndromes). Each item can be rated on a five-point Likert scale. The test is viewed as reliable (test-retest reliability on clinical samples of *r* = .94; [62]) and valid [61]. The Patient Health Questionnaire, German version (PHQ 9; [63]) consists of nine questions regarding depression, with each question targeting one of the nine DSM-IV criteria for the diagnosis “Major Depression”. The test shows high internal consistency (Cronbach’s α=.88; [64]) and seems valid (test sensitivity = .80 when validated against major depressive disorder; [65]).

### Data collection and security protection

All self-report questionnaires for T0, T1, and T2 were filled out online. Access to the data was exclusive to study personnel and investigators. Anonymity was protected at the practitioners’ level by the professional laws binding to secrecy (Austrian Psychologists’ Act and German Psychotherapists’ Act) and at the research level through individual codes for each subject, which makes the data not assignable to a specific participant. Personal data is only accessible through a password-protected table on an equally protected laptop.

### Statistical analysis

First, a descriptive analysis was performed, presenting means and standard deviations of the two treatment conditions for each of the dependent variables for all three time points (pre, post, follow-up). Furthermore, baseline comparisons have been conducted for the nine dependent variables as well as for the demographic variables age and gender. For each variable, either a *t*-test or a $\chi^{2}$-test was used to investigate significant differences at baseline level. Moreover, the data was looked at separately for each therapist group to examine differences between the clusters. The question whether the two treatment conditions differ over time was analyzed using a linear mixed model (LMM) approach. For each variable of interest, a LMM analysis was conducted. The models include random intercepts for subjects and therapists. To account for the data hierarchy, subjects are nested within therapists. A recommended correction for safeguarding against type I error inflation when analyzing clustered data with a small to medium number of clusters is to adjust the degrees of freedom [66]. In this analysis, the Satterthwaite degrees of freedom correction was used [67]. The R packages lme4 and lmerTest were used to conduct the LMM analysis [68]. For effect size calculation, the approach proposed by [69] for calculating Cohen’s *d* was used. According to Cohen, effect sizes can be interpreted using the following conventional cut-off values: d = 0.2 as small, d = 0.5 as medium, and d = 0.8 as large effects [70].

## Results

### Descriptive statistics

Table 5 presents a comprehensive overview, delineating the means and standard deviations for pre, post, and follow-up measurements. These encompass primary outcomes such as anxiety levels (Fear Questionnaire (FQ), Generalized Anxiety Disorder (GAD), Beck Anxiety Inventory (BAI), Panic and Agoraphobia Scale (PAS)), subjective happiness (Flourishing Scale (FS), Satisfaction with Life Scale (SWLS), Positive Psychotherapy Inventory (PPTI), and general psychological distress (ICD-10 Symptom Rating (ISR), Patient Health Questionnaire (PHQ9)) as a secondary outcome across both treatment groups. Upon scrutinizing the means, a notable contrast emerges between pre and post measurements in both groups for all nine variables. A parallel pattern is evident in both groups, indicating a significant change over time. However, no significant disparity is discerned between post-intervention and follow-up periods within each respective group.

**Table 5:** Means and standard deviations for all outcome variables in pre-, post-, and follow-up measurement points.

	Group	Pre		Post		Follow-up	
		M	SD	M	SD	M	SD
**Primary outcomes**							
**Level of anxiety**							
FQ	PPT	21.64	9.30	15.34	9.91	14.77	9.91
	CBT	17.26	9.98	9.98	7.09	10.68	7.94
GAD7	PPT	12.98	4.08	9.36	4.44	9.00	5.10
	CBT	11.75	4.07	7.60	3.43	7.81	4.02
BAI	PPT	27.96	11.20	19.17	12.65	17.17	11.54
	CBT	27.47	11.55	16.89	9.87	16.58	9.81
PAS	PPT	22.92	9.91	15.74	9.43	15.40	10.42
	CBT	20.95	10.45	12.93	8.39	12.11	9.60
**Subjective experience of happiness**							
FS	PPT	38.64	8.87	41.02	8.60	40.77	9.00
	CBT	39.51	8.60	42.46	8.46	43.63	8.34
SWLS	PPT	20.28	6.37	22.17	7.19	22.70	6.01
	CBT	21.39	6.21	23.19	6.71	24.40	6.81
PPTI	PPT	86.92	14.26	93.26	13.53	91.32	17.50
	CBT	89.37	12.80	92.95	13.23	95.30	14.17
**Secondary outcomes**							
**General psychological distress**							
ISR	PPT	46.08	14.88	35.75	15.99	34.79	18.61
	CBT	40.96	13.35	31.79	13.06	31.00	15.72
PHQ9	PPT	11.49	5.41	9.06	5.07	9.66	5.98
	CBT	10.09	5.02	7.58	4.47	7.77	4.95

FQ: Fear Questionnaire, GAD7: Generalized Anxiety Disorder, BAI: Beck Anxiety Inventory, PAS: Panic and Agoraphobia Scale, FS: Flourishing Scale, SWLS: Satisfaction with Life Scale, PPTI: Positive Psychotherapy Inventory, IRS: ICD-10 Symptom Rating, PHQ9: Patient Health Questionnaire.

### Clustered by therapists

**Fig 2:**
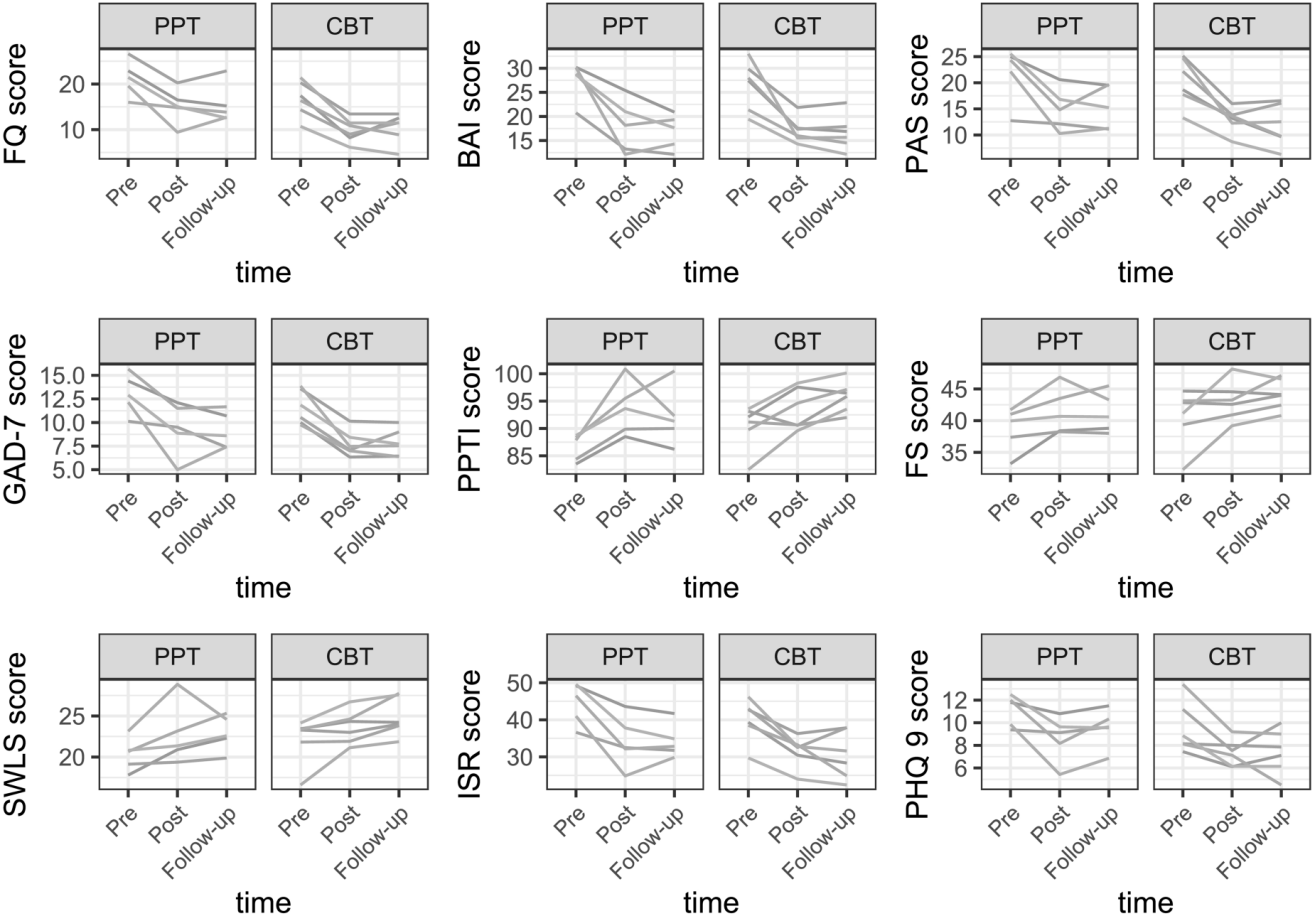
Behavior of each therapist cluster over time for each outcome variable.

The participants received treatment from different pairs of therapists. There were a total of eight therapeutic pairs, with each cluster comprising between six and 22 individuals per treatment group. Fig 2 illustrates the graphical analysis, depicting the behavior of each cluster over time for each outcome variable. While there is a significant difference in intercepts between clusters for most outcome variables, it seems that these differences do not vary greatly over time. The slopes of the clusters demonstrate consistent behavior over time, with minimal variance. This consistency is reflected in the similarity of their respective slopes, suggesting a notable degree of similarity in their temporal patterns.

### Primary outcomes

#### Level of anxiety.

The *F*-tests assessing interaction effects for FQ (*F*(2, 216)=.38, *p* = .682), BAI (*F*(2, 216)=.53, *p* = .592), PAS (*F*(2, 216)=.36, *p* = .699), and GAD7 (*F*(2, 216)=.30, *p* = .74) yielded *p*-values surpassing the threshold of .05. These findings are detailed in Table 6. The LMM analyses revealed no significant interaction effects between treatment and time for any primary outcome (FQ, BAI, PAS, GAD-7). This indicates that the change in anxiety symptoms over time did not differ significantly between the CBT and PPT groups. However, there was a significant main effect of time across all outcomes: anxiety symptoms decreased during treatment and remained reduced at follow-up. A main effect of group was found only for the FQ, with participants in the CBT condition reporting significantly lower fear levels than those in the PPT condition (b=−4.46, *p* = .017, d=−.49).

**Table 6:** *F*-tests for FQ, BAI, PAS and GAD-7.

	FQ				BAI				PAS				GAD7			
Factor	df1	df2	*F*	*p*	df1	df2	*F*	*p*	df1	df2	*F*	*p*	df1	df2	*F*	*p*
group	1	102.00	8.72	0.006	1	102.00	0.33	0.565	1	102.00	2.49	0.117	1	102.00	3.86	0.066
time	2	216.00	52.86	<0.001	2	216.00	74.15	<0.001	2	216.00	67.75	<0.001	2	216.00	60.75	<0.001
group:time	2	216.00	0.38	0.682	2	216.00	0.53	0.592	2	216.00	0.36	0.699	2	216.00	0.30	0.74
gender	1	104.34	0.07	0.797	1	106.00	0.30	0.583	1	106.00	1.75	0.189	1	102.74	0.14	0.712
age	1	105.21	0.08	0.777	1	106.00	0.27	0.605	1	106.00	4.65	0.033	1	103.94	0.43	0.511

FQ: Fear Questionnaire, BAI: Beck Anxiety Inventory, PAS: Panic and Agoraphobia Scale, GAD7: Generalized Anxiety Disorder.

**Fig 3:**
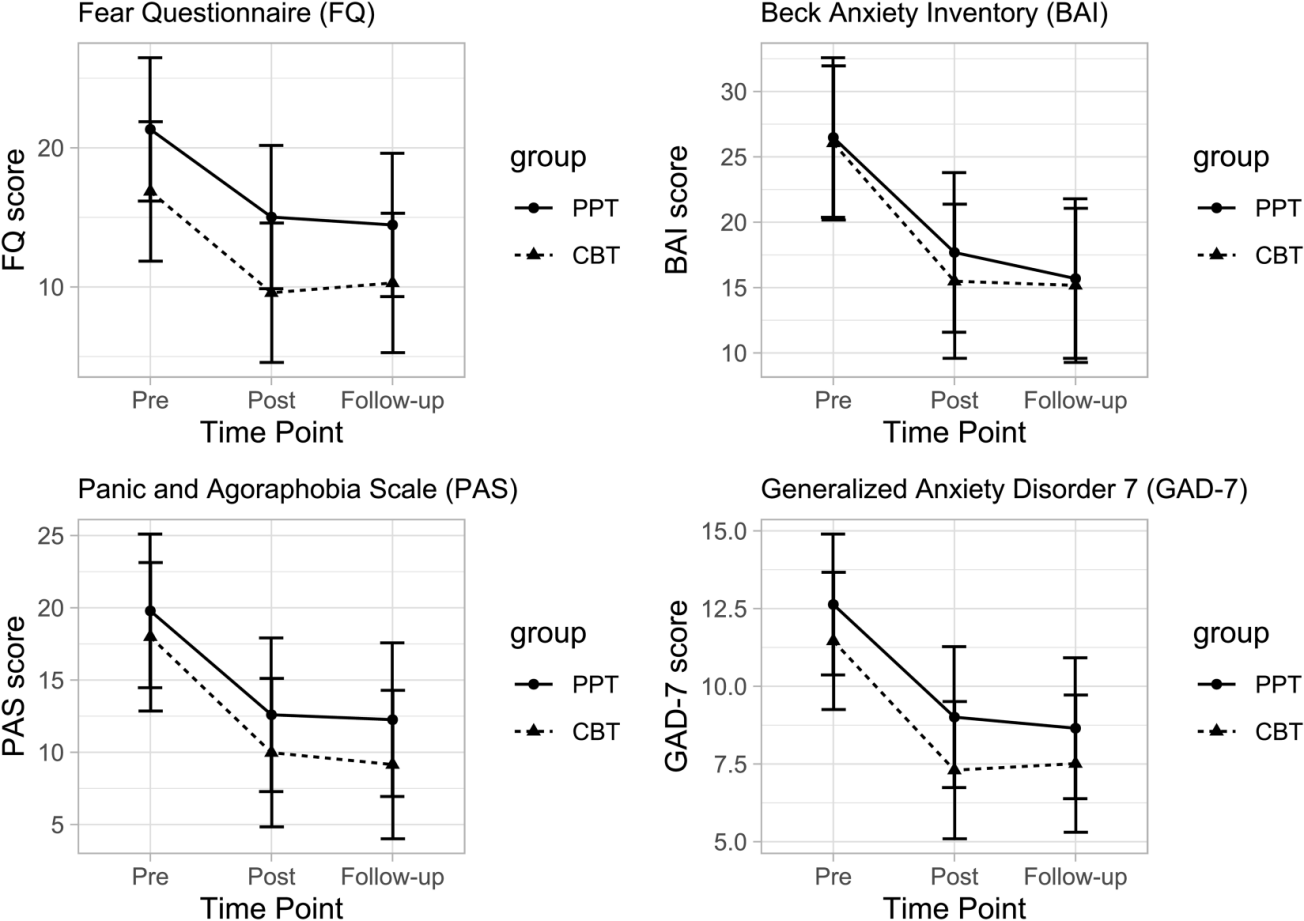
Plots of means for FQ, BAI, PAS, and GAD-7.

The interaction of group[CBT] × time[post] as well as group[CBT] × time[follow-up], instead, do not show significant differences across all primary outcomes. This shows that the improvement with time does not depend on the assigned group. All results are adjusted for gender and age. A comprehensive summary of the Linear Mixed Model (LMM) results is presented in Tables 7 and 8, and in Fig 3.

**Table 7:** Linear mixed models for FQ, BAI, PAS, GAD-7.

Predictors	FQ	BAI	PAS	GAD7
	Estimates	Estimates	Estimates	Estimates
(Intercept)	19.88*** (5.53)	23.09*** (6.66)	9.78 (5.83)	11.41*** (2.40)
group [CBT]	−4.46* (1.81)	−0.42 (2.13)	−1.79 (1.83)	−1.17 (0.83)
time [post]	−6.30*** (1.09)	−8.79*** (1.41)	−7.19*** (1.13)	−3.62*** (0.59)
time [follow-up]	−6.87*** (1.09)	−10.79*** (1.41)	−7.53*** (1.13)	−3.98*** (0.59)
gender [female]	1.19 (4.60)	3.06 (5.56)	6.44 (4.87)	0.74 (2.00)
age	0.02 (0.09)	0.05 (0.10)	0.20* (0.09)	0.02 (0.04)
group [CBT] × time [post]	−0.98 (1.52)	−1.79 (1.96)	−0.83 (1.57)	−0.54 (0.82)
group [CBT] × time [follow-up]	0.29 (1.52)	−0.10 (1.96)	−1.31 (1.57)	0.03 (0.82)
**Random Effects**				
$\sigma^{2}$	31.64	52.53	33.82	9.30
$\tau_{00}$	50.51 subj_ID:th_ID	72.38 subj_ID	57.69 subj_ID	8.39 subj_ID:th_ID
	1.49 th_ID			0.22 th_ID
ICC	0.62	0.58	0.63	0.48
N	110 subj_ID	110 subj_ID	110 subj_ID	110 subj_ID
	8 th_ID			8 th_ID
Observations	330	330	330	330

FQ: Fear Questionnaire, BAI: Beck Anxiety Inventory, PAS: Panic and Agoraphobia Scale, GAD7: Generalized Anxiety Disorder.

**Table 8:** Effect sizes of main effects and interactions for FQ, BAI, PAS, GAD7.

Predictors	FQ	BAI	PAS	GAD7
	d	d	d	d
group [CBT]	−0.49	−0.04	−0.19	−0.28
time [post]	−0.69	−0.79	−0.75	−0.86
time [follow-up]	−0.75	−0.97	−0.79	−0.94
group [CBT] × time [post]	−0.11	−0.16	−0.09	−0.13
group [CBT] × time [follow-up]	0.03	−0.01	−0.14	0.01

FQ: Fear Questionnaire, BAI: Beck Anxiety Inventory, PAS: Panic and Agoraphobia Scale, GAD7: Generalized Anxiety Disorder.

#### Subjective experience of happiness.

The analysis of indicators related to the subjective experience of happiness, conducted using a LMM, aligns with the findings related to the extent of anxiety. All three outcomes fail to reveal a significant interaction effect between treatment conditions and temporal factors. In the PPTI (*F*(2, 216) = 2.06, *p* = .13), the FS (*F*(2, 216.01) = 1.17, *p* = .313), and the SWLS (*F*(2, 216) = .27, *p* = .765), individuals undergoing PPT did not display significantly higher or lower levels over time compared to those in the CBT group. The corresponding effects are presented in Table 9.

**Table 9:** *F*-tests for PPTI, FS, SWLS.

Factor	PPTI				FS				SWLS			
	df1	df2	*F*	*p*	df1	df2	*F*	*p*	df1	df2	*F*	*p*
group	1	106.00	0.70	0.404	1	106.00	1.07	0.308	1	106.00	1.43	0.235
time	2	216.00	14.88	<0.001	2	216.01	12.64	<0.001	2	216.00	14.81	<0.001
group:time	2	216.00	2.06	0.130	2	216.01	1.17	0.313	2	216.00	0.27	0.765
gender	1	106.00	0.67	0.414	1	104.26	0.17	0.680	1	106.00	1.10	0.296
age	1	106.00	5.04	0.027	1	105.22	3.71	0.057	1	106.00	0.09	0.762

PPTI: Positive Psychotherapy Inventory, FS: Flourishing Scale, SWLS: Satisfaction with Life Scale.

The LMM analyses revealed a significant main effect of time across all outcomes: the subjective experience of happiness increase during treatment and remained high at follow-up. The main effect of group was not significant for any of the outcome variables. This means that, across all time points, there were no overall differences between the groups. The interaction of group[CBT] × time[post] as well as group[CBT] × time[follow-up] do not show significant differences across all outcomes (PPTI, FS, SWLS). This shows that the improvement with time does not depend on the assigned group.

**Fig 4:**
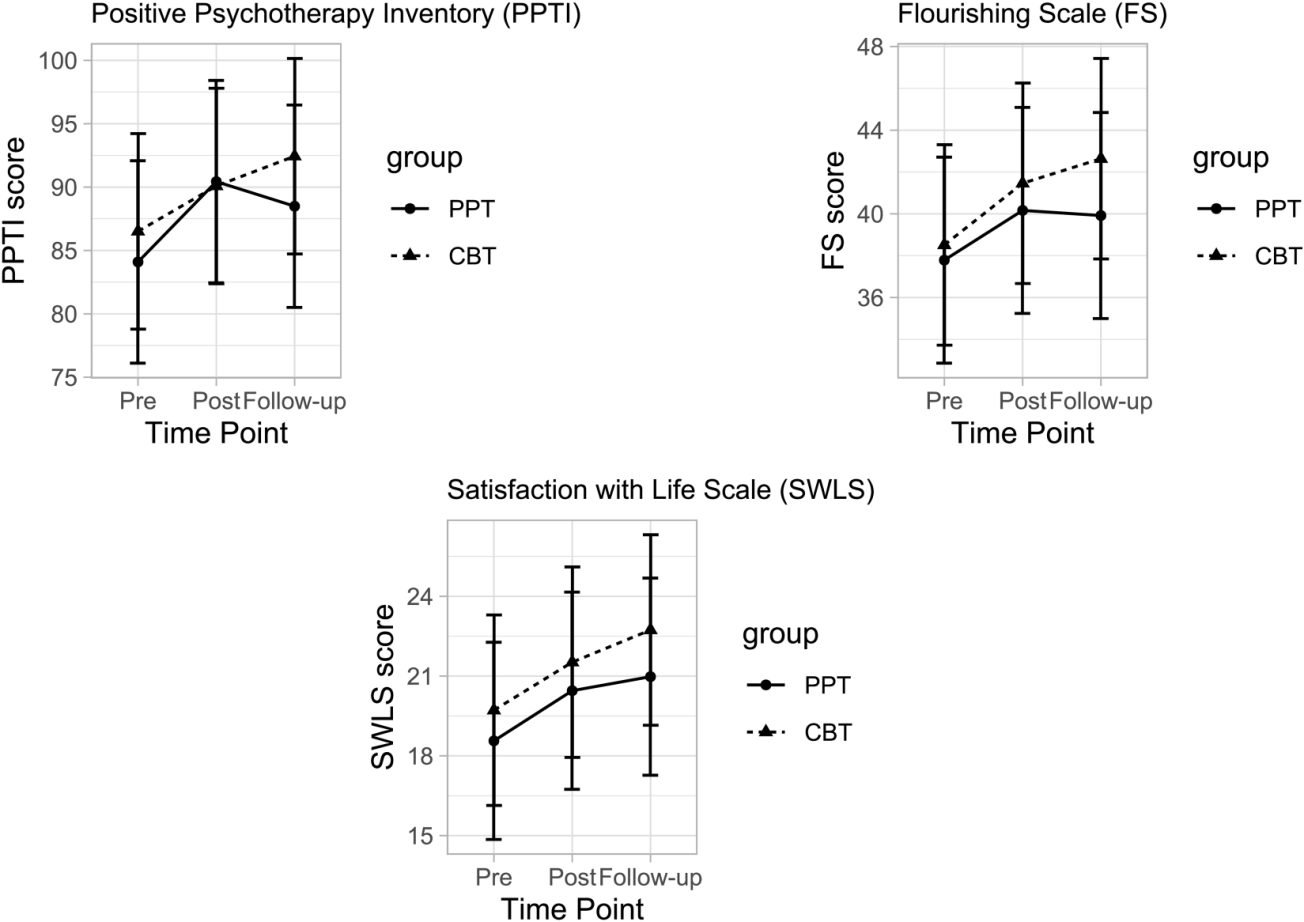
Plots of means for PPTI, FS, SWLS.

All reported results have been adjusted for the covariates of gender and age. Comprehensive details of the results can be found in Tables 10 and 11, and in Fig 4.

**Table 10:** Linear mixed models for PPTI, FS, SWLS.

Predictors	PPTI	FS	SWLS
	Estimates	Estimates	Estimates
(Intercept)	91.75 *** (8.79)	42.41 *** (5.30)	17.44 *** (4.08)
group [CBT]	2.41 (2.70)	0.73 (1.71)	1.15 (1.26)
time [post]	6.34 *** (1.54)	2.38 * (0.97)	1.89 * (0.73)
time [follow-up]	4.40 ** (1.54)	2.13 * (0.97)	2.42 ** (0.73)
gender [female]	6.02 (7.35)	1.82 (4.41)	3.58 (3.41)
age	−0.31 * (0.14)	−0.16 (0.08)	−0.02 (0.06)
group [CBT] × time [post]	−2.76 (2.14)	0.57 (1.34)	−0.08 (1.02)
group [CBT] × time [follow-up]	1.53 (2.14)	1.99 (1.34)	0.60 (1.02)
**Random Effects**			
$\sigma^{2}$	63.13	24.75	14.28
$\tau_{00}$	136.05 subj_ID	47.89 subj_ID:th_ID	29.05 subj_ID
		1.46 h_ID	
ICC	0.68	0.67	0.67
N	110 subj_ID	110 subj_ID	110 subj_ID
		8 th_ID	
Observations	330	330	330

PPTI: Positive Psychotherapy Inventory, FS: Flourishing Scale, SWLS: Satisfaction with Life Scale.

**Table 11:** Effect sizes of main effects and interactions for PPTI, FS, SWLS.

Predictors	PPTI	FS	SWLS
	d	d	d
group [CBT]	0.17	0.08	0.18
time [post]	0.45	0.28	0.29
time [follow-up]	0.31	0.25	0.37
group [CBT] × time [post]	−0.20	0.07	−0.01
group [CBT] × time [follow-up]	0.11	0.23	0.09

PPTI: Positive Psychotherapy Inventory, FS: Flourishing Scale, SWLS: Satisfaction with Life Scale.

### Secondary outcomes

#### General psychological distress.

The exploration of psychological distress indicators using a LMM aligns with the patterns observed in earlier outcomes. A thorough analysis did not reveal any significant interaction effect between treatment and time concerning the ISR or PHQ-9, as indicated by (*F*(2, 216) = .13, *p* = .877) and (*F*(2, 216) = .17, *p* = .845). This suggests that individuals undergoing PPT did not exhibit significantly higher or lower levels of psychological distress over time compared to those in the CBT group. However, the main effect of time was statistically significant in both outcome measures. The corresponding effects are presented in Table 12.

**Table 12:** *F*-tests for ISR, PHQ9.

Factor	ISR				PHQ9			
	df1	df2	*F*	*p*	df1	df2	*F*	*p*
group	1	106.00	3.04	0.084	1	106.00	3.78	0.055
time	2	216.00	35.51	<0.001	2	216.00	17.34	<0.001
group:time	2	216.00	0.13	0.877	2	216.00	0.17	0.845
gender	1	106.00	0.14	0.705	1	106.00	1.65	0.202
age	1	106.00	2.29	0.134	1	106.00	2.05	0.155

IRS: ICD-10 Symptom Rating, PHQ9: Patient Health Questionnaire.

An in-depth analysis elucidated that there were no notable differences between the groups in values of psychological distress for ISR and PHQ-9. In both outcome variables, participants showed significantly lower values in post and follow-up measurements compared to pre-measurement.

**Fig 5:**
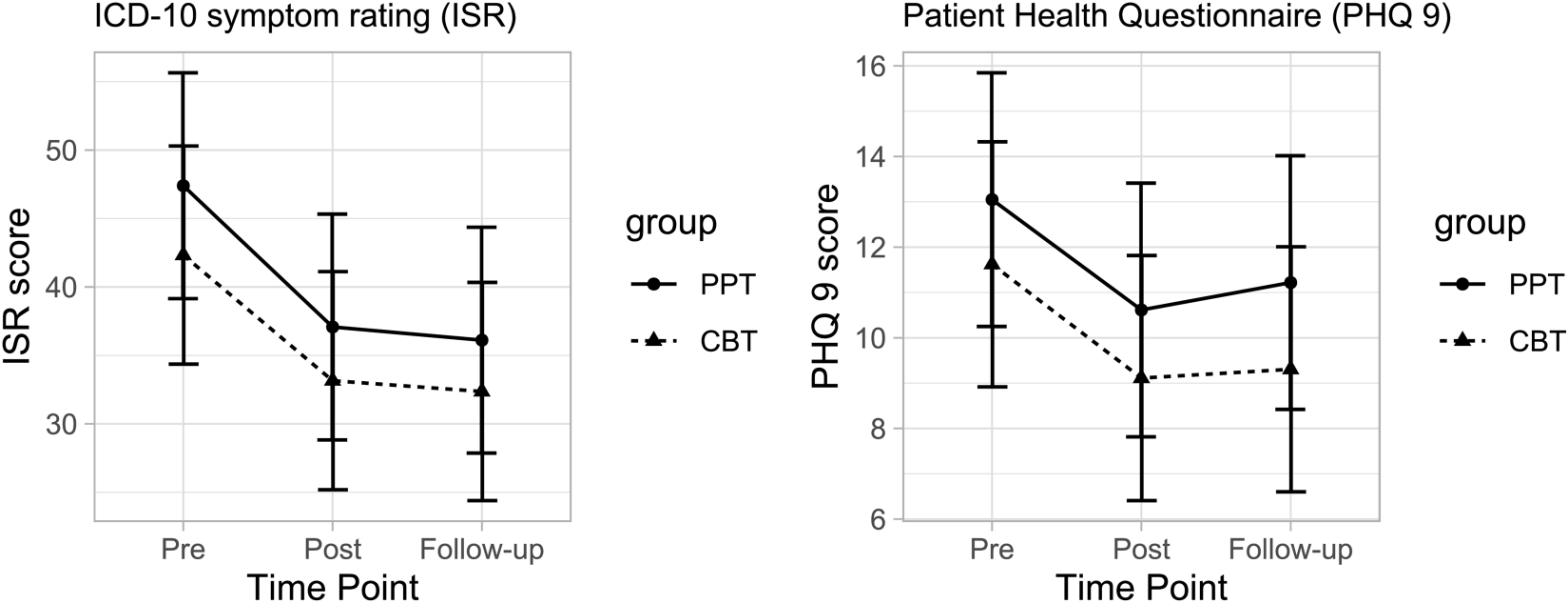
Plots of means for ISR, PHQ9.

As in previous results, the general psychological distress variables showed almost parallel slope patterns in the graphs. Both groups exhibited a strong decline between pre and post measurement and an almost stable behavior between post and follow-up measurement. Again, all results have been adjusted for gender and age covariates. Comprehensive details of the results can be found in Tables 13 and 14, and in Fig 5.

**Table 13:** Linear mixed models for ISR, PHQ9.

Predictors	ISR	PHQ9
	Estimates	Estimates
(Intercept)	41.51 *** (8.97)	12.32 *** (3.05)
group [CBT]	−5.07 (2.93)	−1.43 (0.98)
time [post]	−10.32 *** (2.02)	−2.43 *** (0.65)
time [follow-up]	−11.28 *** (2.02)	−1.83 ** (0.65)
gender [female]	−2.84 (7.48)	−3.27 (2.55)
age	0.21 (0.14)	0.07 (0.05)
group [CBT] × time [post]	1.15 (2.80)	−0.07 (0.90)
group [CBT] × time [follow-up]	1.32 (2.80)	−0.49 (0.90)
**Random Effects**		
$\sigma^{2}$	107.60	11.16
$\tau_{00}$	126.89 subj_ID	15.17 subj_ID
ICC	0.54	0.58
N	110 subj_ID	110 subj_ID
Observations	330	330

IRS: ICD-10 Symptom Rating, PHQ9: Patient Health Questionnaire.

**Table 14:** Effect sizes of main effects and interactions for ISR, PHQ9.

Predictors	ISR	PHQ9
	d	d
group [CBT]	−0.33	−0.28
time [post]	−0.67	−0.47
time [follow-up]	−0.74	−0.36
group [CBT] × time [post]	0.07	−0.01
group [CBT] × time [follow-up]	0.09	−0.09

IRS: ICD-10 Symptom Rating, PHQ9: Patient Health Questionnaire.

## Discussion

The aim of the present study was to investigate the effectiveness of PPT in reducing anxiety symptoms and general psychological distress, as well as in enhancing happiness and quality of life, based on the assumption that individuals can be effectively treated by strengthening positive psychological resources, in a large sample of German-speaking individuals with anxiety disorders. The results of the study showed that the primary outcome extent of anxiety decreased significantly in all anxiety-measuring questionnaires (BAI, PAS, GAD-7, FQ) pre- to post-treatment, with moderate to large effect sizes (d=−.69 to −.86). The reduction in the scores was stable up to three months later at the follow-up. These results are in line with previous related studies showing that PPT or PPTI can be effective in reducing anxiety in patients with asthma [71] and medically ill patients [72], with the improvement of the anxiety scores being maintained up to several weeks compared to a control group. Similarly, the scores on the tests for subjective feeling of happiness (PPTI, FS) and quality of life (SWLS) improved significantly over the course of the therapy (pre vs. post intervention) with small effect sizes (*d* = .28 to .45) and stayed at a consistent level until the follow-up. This supports existing literature showing that positive group-psychotherapy leads to significantly better quality of life in the intervention group compared to the control group [73]. Additionally, a meta-analysis indicates that PPTI can alleviate subjective and psychological well-being up to six months after the intervention [74]. The results in terms of subjective happiness and quality of life were similarly high in both intervention groups. A possible explanation for that could be that the quality of life and the feeling of happiness increase independently of the form of therapy. According to the current literature, therapy with CBT also has a positive effect on satisfaction and happiness [75,76]. Additionally, with the reduction of the level of the patients’ anxiety, they will feel happier and in turn experience an improved quality of life. It is also worth considering that the relatively high baseline level of distress and symptom burden may have increased the likelihood of detecting significant change across all measured outcomes. Furthermore, the shared group format for both interventions could have contributed additional therapeutic benefits, such as peer support, social connectedness, and group cohesion, which are known to enhance treatment adherence and psychological well-being. These nonspecific factors may help explain why both PPT and CBT demonstrated comparable levels of improvement across domains.

The same results were found for the secondary outcome measure general level of psychological distress (ISR, PHQ-9), showing lower scores post- vs pre-intervention (d=−.67 & −.47) as well as at follow-up compared to pre-intervention (d=−.36 & −.74). These findings were seen in both intervention groups, PPT and CBT. This is the first study to compare the effectiveness of PPT for anxiety disorders with CBT with a large sample in an online group setting. To date, CBT has been the standard treatment for anxiety along with pharmacotherapy [2], with moderate to high effects from pre- to post-treatment ([77] *g* = .73 to 1.58) when compared to placebo or waitlist control groups [78,79]. Whether PPT can be compared to or is even better than CBT in terms of success rate was the aim of the present study. The results clearly indicate the efficacy of PPT in the treatment of anxiety, with effects lasting up to at least three months. PPT does indeed appear to be comparable to CBT in terms of treatment success, with small effect sizes in the questionnaires for the main effects regarding the outcome measure level of anxiety (d=−.49 to −.04; Table 8) as well as the outcome measure subjective experience of happiness (*d* = .08 to .18; Table 11), and the secondary outcome measure general psychological distress (d=−.33 to −.28; Table 14). This supports previous studies regardin*g* PPT which showed its positive impact on well-being [14], anxiety [18], and depression [13,15].

The sustained reduction of anxiety symptoms and improvements in well-being observed in this study may be attributed to mechanisms that are common across therapeutic approaches. Recent advances in transdiagnostic interventions emphasize the importance of targeting shared vulnerability factors such as emotion dysregulation, maladaptive cognitive patterns, and avoidance behaviors [80–83]. It is plausible that both PPT and CBT exert their effects through modulation of these processes, thereby explaining their comparable effectiveness in treating anxiety disorders. Additionally, the observed improvements in happiness and quality of life may reflect a general enhancement of psychological resilience and adaptive functioning, which is consistent with findings from transdiagnostic psychiatry.

Moreover, the online group format utilized in this study may represent an important innovation in the delivery of mental health interventions. Given the growing demand for scalable and accessible treatment options, particularly in the wake of the COVID-19 pandemic, the demonstration of effectiveness for both PPT and CBT in an online setting is highly relevant. This aligns with broader trends in digital mental health, which emphasize the importance of evidence-based, low-threshold interventions that can reach underserved populations.

The follow-up data showing maintained or even enhanced gains three months post-treatment suggest that participants may continue to apply therapeutic principles autonomously after the intervention has ended.

In addition, individual difference variables such as baseline severity, personality traits, or motivation for treatment could play a role in moderating treatment outcomes. Future analyses should examine whether certain subgroups benefit more from PPT or CBT, potentially paving the way for more personalized approaches in therapy selection. The present study makes a significant contribution to the field, as it is—to our knowledge—the largest randomized controlled trial to date comparing PPT and CBT in the treatment of anxiety disorders in an online group format (*n* = 110). It provides robust evidence on the effectiveness of both approaches and offers valuable insights for the advancement of evidence-based digital mental health interventions. This study not only confirms the therapeutic value of PPT in reducing anxiety symptoms and enhancing well-being but also suggests that it may offer an alternative approach to CBT in online settings. Continued research is needed to refine treatment protocols, explore underlying mechanisms of change, and determine optimal conditions for dissemination and implementation. These further studies should be adequately powered and include formal non-inferiority or equivalence designs, to determine the comparative effectiveness of PPT more reliably.

### Limitations

Several study limitations must be acknowledged. A major limitation of the present study is the pronounced gender imbalance of the sample, with a substantial predominance of female participants. Although this is partly consistent with the higher prevalence of anxiety disorders among women [84,85] and their generally greater utilization of mental health services [86–89], the proportion of female participants substantially exceeded epidemiological expectations. This imbalance represents a significant threat to the external validity of the findings. Accordingly, the results must be interpreted with considerable caution, as their generalizability to more gender-balanced samples and especially to predominantly male populations is likely to be markedly restricted. It must also be criticized that the therapy was carried out by nine different therapists, which makes comparisons challenging. In order to minimize the subjective influence of the therapists, adherence to the manual was checked through regular intervision and supervision. A third-party assessment of the therapists with regard to the outcomes would also have been of interest and should be taken into account in future studies. External evaluations are crucial in psychotherapeutic processes for several reasons. First, they are a separate source of information, which offers an objective perspective. They can be utilized when the individual under investigation is either unwilling or unable to provide sufficient information. Second, external evaluations can validate the progress and outcomes of therapy, providing measurable evidence of improvement or the need for adjustments. Another limitation of the study, the dropout rate, should also be mentioned here. There was a loss of subjects and missing data in both groups: 18% dropped out at post-treatment and 21% dropped out during the three-month follow-up. However, it should be noted that the dropouts were almost evenly distributed across the groups and that LMM are robust to missing data [90]. One possible reason for the number of dropouts could be the inhomogeneity of anxiety disorders in the groups. Very different reasons were given for dropping out (e.g., illness in the family, commitment to individual therapy). A notable limitation of the study is that depressive symptoms were not systematically assessed; including such measures could have provided a more comprehensive understanding of the intervention’s effects, especially given the close relationship between anxiety and depression. We therefore recommend that future studies incorporate assessments of depressive symptoms to further clarify these interrelations.

### Conclusion

In summary, the present study showed that positive psychology yields the same results as the standardized CBT interventions, which is the gold standard in the treatment of anxiety disorders. Both treatment modalities significantly reduced anxiety symptoms and increased the measured positive outcomes. However, since the study sample consisted almost exclusively of women, future studies should focus on male participants for a better understanding of the efficacy of PPT on both genders. Furthermore, to generalize the effectiveness of PPT to other mental disorders, additional research is strongly recommended. Additionally, an investigation of the long-term sustainability (longer than three months) of these effects is needed.

## Supporting information

S1 Appendix Study protocol German. Study protocol as approved by the ethics committee in original language (German).
(PDF)

S2 Appendix Study protocol English. English translation of the study protocol as approved by the ethics committee.
(PDF)

S3 Appendix CONSORT checklist. (PDF)

S4 Appendix Ethics approval letter (German). Original approval letter of the ethics committee.
(PDF)

S5 Appendix Ethics approval letter (English). English translation of the approval letter of the ethics committee.
(PDF)

S6 Dataset Raw Data. Complete set of collected data collected during the study.
(SAV)
