# Supplementary material for: Cognitive Behavior Therapy (CBT) and Positive Psychotherapy (PPT) for the treatment of anxiety disorders in an online group setting: A randomized controlled trial
Source: PLoS One. 2026 Aug 12;21(8):e0355614. doi: 10.1371/journal.pone.0355614 (PMC13466016; doi:10.1371/journal.pone.0355614)
Supplement: Supplementary file 4 [file pone.0355614.s004.pdf]

PARIS LODRON UNIVERSITÄT SALZBURG

ETHIKKOMMISSION
Paris Lodron-Universität Salzburg

Univ.-Prof.
Dr. Frank WILHELM
Vorsitzender

Kapitelgasse 4-6
A-5020 Salzburg | Austria
Tel.: +43 / (0) 662 / 8044 - 5119
frank.wilhelm@sbg.ac.at
www.uni-salzburg.at/ethikkommission

Salzburg, 6.7.2021
EK-GZ 22/2021

VOTUM DER ETHIKKOMMISSION

Arbeitstitel: *Online-Interventionsstudie bei Angststörungen mit Kognitiver Verhaltenstherapie und Positiver Psychologie*

Antragstellerin: Catiana Engelhardt

Nach Beratung der Ethikkommission der Paris Lodron-Universität Salzburg am 2. Juni 2021 ergeht folgender Beschluss:

Es besteht kein Einwand gegen die Durchführung der Studie.

Bei unerwarteten körperlichen und psychischen Reaktionen ist die Studie auszusetzen und die Ethikkommission darüber zu informieren.

Bei jeglichen Änderungen z.B. in der Themenstellung, der verwendeten Methoden oder des Proband*innenkreises ist die Ethikkommission erneut zu befassen.

Wir wünschen Ihnen eine erfolgreiche Durchführung des Projekts und weisen grundsätzlich darauf hin, dass die ethische und rechtliche Verantwortung für die Durchführung der Studie unabhängig von der positiven Erklärung der Kommission bei der Leiterin bzw. dem Leiter des Projekts sowie den Projektmitarbeiter*innen verbleibt.

Univ.-Prof. Dr. Frank WILHELM
Vorsitzender der Ethikkommission
