# Supplementary material for: Cognitive Behavior Therapy (CBT) and Positive Psychotherapy (PPT) for the treatment of anxiety disorders in an online group setting: A randomized controlled trial
Source: PLoS One. 2026 Aug 12;21(8):e0355614. doi: 10.1371/journal.pone.0355614 (PMC13466016; doi:10.1371/journal.pone.0355614)
Supplement: Supplementary file 5 [file pone.0355614.s005.pdf]

ETHIKKOMMISSION

Paris Lodron-Universität Salzburg

Univ.-Prof.

Dr. Frank WILHELM

Vorsitzender

Kapitelgasse 4-6
A-5020 Salzburg | Austria
Tel.: +43 / (0) 662 / 8044 - 5119

frank.wilhelm@sbg.ac.at

www.uni-salzburg.at/ethikkommission

Salzburg, 6.7.2021
EK-GZ 22/2021

VOTE OF THE ETHICS COMMITTEE

Title: Online intervention study in anxiety disorders with Cognitive Behavioral Therapy and Positive Psychology.

Applicant: Catiana Engelhardt

After deliberation of the Ethics Committee of Paris Lodron University Salzburg on June 2, 2021, the following resolution is issued:

There is no objection to the conduct of the study.

In case of unexpected physical and psychological reactions, the study has to be suspended and the ethics committee has to be informed.

In the event of any changes, e.g. in the subject matter, the methods used or the group of subjects, the ethics committee must be consulted again.

We wish you a successful implementation of the project and point out that the ethical and legal responsibility for the implementation of the study remains with the head of the project and the project staff, regardless of the positive declaration of the committee.

Prof. Dr. Frank WILHELM
Chairman of the Ethics Committee
